# Impact of receptor clustering on ligand binding

**DOI:** 10.1186/1752-0509-5-48

**Published:** 2011-03-31

**Authors:** Bertrand R Caré, Hédi A Soula

**Affiliations:** 1 Université de Lyon, Laboratoire d'InfoRmatique en Image et Systèmes d'information, CNRS UMR5205, F-69621, France; 2 Université de Lyon, Cardiovasculaire Métabolisme et Nutrition, Inserm UMR1060, F-69621 Villeurbanne Cédex, France; 3 EPI BEAGLE, INRIA Rhône-Alpes, 69603 Villeurbanne, France

## Abstract

**Background:**

Cellular response to changes in the concentration of different chemical species in the extracellular medium is induced by ligand binding to dedicated transmembrane receptors. Receptor density, distribution, and clustering may be key spatial features that influence effective and proper physical and biochemical cellular responses to many regulatory signals. Classical equations describing this kind of binding kinetics assume the distributions of interacting species to be homogeneous, neglecting by doing so the impact of clustering. As there is experimental evidence that receptors tend to group in clusters inside membrane domains, we investigated the effects of receptor clustering on cellular receptor ligand binding.

**Results:**

We implemented a model of receptor binding using a Monte-Carlo algorithm to simulate ligand diffusion and binding. In some simple cases, analytic solutions for binding equilibrium of ligand on clusters of receptors are provided, and supported by simulation results. Our simulations show that the so-called "apparent" affinity of the ligand for the receptor decreases with clustering although the microscopic affinity remains constant.

**Conclusions:**

Changing membrane receptors clustering could be a simple mechanism that allows cells to change and adapt its affinity/sensitivity toward a given stimulus.

## Background

The binding kinetics between cell surface receptors and extracellular biomolecules are critical to all intracellular and intercellular activity. Modelling and predicting of receptor-mediated cell functions are facilitated by measurement of the binding properties on whole cells. Therefore, these measurements, however elaborate, have been based on the ground of chemical enzyme/substrate formalism [1-4]. Such formulations were derived from the law of mass-action that evaluates local reaction rates from averaged chemical species densities over the medium volume. Mass-action laws are mean-field approximations because they evaluate local reaction rates on the basis of average values of the reactant density over a large spatial domain. In addition, it amounts to assume that ligand/receptor interactions are independent [5,6].

These assumptions may fail in real biological systems, in particular considering membrane receptors which are restricted to only 2 of the 3 spatial dimensions [7,8]. The effect of binding kinetics for membrane-restricted receptors (on spherical cells) has already been investigated by Berg and Purcell [9]. This study focused on the spatial restriction of receptors to a 2D support while interacting with bulk ligand diffusing in a 3 D medium, and resulted in an expression for reaction rate coefficients as non-linear functions of cell surface receptor density. This pioneer study has been enriched by further works towards reversibility and rebinding [10], receptor density [11], time dependency [12], and gradient sensing capabilities [13,14]. Taking a step further, the spatial organization of receptors on the membrane itself should also be taken into account. At first glance, since membrane receptors are bound to the cell membrane that allows a lateral degree of freedom, one would expect a simple (and homogeneous) distribution of receptors on the membrane. Indeed, cell membrane is composed of a mixture of phospholipids in a fluid phase and as such, in the classical fluid-mosaic model of membrane [15], membranes components undergo isotropic random movement akin to Brownian motion [16,17]. In this model, the resulting equilibrium distribution of components - among them receptors - is therefore homogeneous. Recently, however, this picture has evolved considerably towards a non-homogeneous distribution of the usual components of cell membranes [18-21]. Indeed, more and more evidence points towards the existence of micro-domains enriched in various lipids such as cholesterol as well as other proteins. In particular, receptor colocalization in lipid rafts and other membrane structures have been reported in cells [22-24].

This localization and clustering may have a dramatic influence on signalling. This influence remains, however, unclear as literature reports contradictory effects of clustering/declustering on signalling (see e.g. [23,25]). This is probably due to the method of destroying cholesterol-rich domains via methyl-*β*-cyclodextrin which may have other effects than simply unclustering membrane receptors, and alter signalling functions.

In any case, the impact of an inhomogeneous receptor density *on *the membrane itself has been only studied recently. Only few theoretical contributions have been reported in some specific cases: : bacteria sensitivity [26] and chemotaxis [27], G-protein activation [28], simple model of trans-phosphorylation (implying two receptors only) [29].

In addition, several more detailed studies illustrate the possible effect of receptor clustering on receptor binding by inducing enhanced rebinding or ligand receptor switching [30-33], or enhancing encounter probability of activated receptors with submembranar signalling proteins such as in GPCR signalling pathways [34].

Notably [32] proposes that clustering provides higher rebinding capabilities and therefore helps to obtain a better response - i.e. more binding events. However, another analysis [35] proposes that the forward rate constant is diminished when receptors are clustered, providing in that case less binding events. Both effects counteract themselves, and the final output remains to be studied.

Considering ligand-receptor binding as a diffusion-limited reaction [9,10], we investigated how receptor distribution may impact this primordial step of signalling, ligand binding to receptor extracellular domain. We will restrict ourselves to ligand-receptor binding probabilistic mechanisms at the early stage of signalling, that is, without considering specific biological/biochemical interactions between receptors themselves, nor between receptors and internal signalling proteins, but only the spatial aspects of ligand-receptor interaction at cell surface. We place this study in the context of generic clustering of receptors that cover the whole cell surface.

In order to investigate the effects of receptor clustering on ligand binding, we present two joint approaches of ligand receptor binding at equilibrium when receptors are organized in clusters at cell surface. We consider three membrane receptor layouts illustrating three degrees of spatial correlation. These layouts, for two of which a simple ODE description is available, are studied in the context of ligand-receptor reversible binding. The three layouts are investigated following computer based simulations conjointly with an ODE formalism, the latter adapted to include spatial characteristics of receptor organization.

Ligands are assumed to diffuse freely above the membrane without interaction except when they can bind stochastically to receptors. Receptors are modelled as still positions on the membrane. Ligand-receptor complex formations are stochastic events occurring whenever a ligand is near enough a free receptor. More precisely, it occurs whenever the ligand lies in a defined area above the receptor position. This area is called the *affinity zone*. This simple binding model can be implemented into both an ODE formalism and computer simulations in to investigate the effects of spatial correlation on total receptor occupation. It allows fast computation and exploration of various receptor configurations together with an analytic formulation of receptor occupation. Using constant reaction rates (which can be easily related to simulation parameters), we compare the amount of complex binding at equilibrium between these different layouts. We show that, contrary to intuition, clustering decreases the overall binding activity: the number of complexes at equilibrium for equal ligand concentration are lower in the clustered case than in the homogeneous case. This drop in the so-called "apparent" affinity increases with clustering as dose-response curves are increasingly shifted to the right.

## Methods

We describe below the three possibilities of spatial correlation we have chosen to investigate. For each, we present the assumptions made in order to model them properly, the simple analytical formulation we derived whenever it was possible, and the corresponding individual-based model used in simulation. As mentioned in introduction, we consider monovalent ligands reversibly binding to monovalent receptors which are independent from each other.

### No spatial correlation

The first layout consists of receptors homogeneously set on the membrane, which stands as a reference configuration of homogeneously spread receptors on the cell membrane. The classical approach to model ligand-receptor interaction is through reaction mechanism akin to enzymatic reactions. In the case of monovalent receptors, the most simple model remains the classical Ligand-Receptor Binding Equilibrium equation:(1)

where *L *will be the ligand and *R *the receptor. When docked, the ligand forms with the receptor a complex *C*. The reaction is reversible with the forward rate constant *k*_1 _and backward rate constant *k*_-1_.

The further steps involve some generally implicit assumptions: the complex concentration variation will be the sum of two parts. The negative rate of complex dissociation will be *k*_-1 _times the complex number. The statistical process underneath this assumption relies basically upon a time independent (exponential) undocking probability [36].

On the other hand, the complex formation equation is based on what is called *the law of mass action *which states that the rate of a reaction is proportional to the product of the concentrations of the reactants. In essence, this law simply states that the reaction rate is proportional to the *rate of encounter *of reactants in the medium. This rate of encounter is itself proportional to the joint probability to find both reactants in the same vicinity. These probabilities are in the case of homogeneous medium the respective concentrations. As [7] have pointed out, this formulation is correct whenever the medium is well-stirred and isotropic with respect to diffusion. In addition, one must assume that particles are independent from each other. Note that in that case, at equilibrium, the relation is well known [5](2)

where lower case indicates quantities of corresponding species. The total number of receptors will be denoted as *r*_0 _and  is the dissociation constant. Variables can be made dimensionless via l* = l/*κ *and c* = *c*/*r*_0_. Note for later that we have two ways to retrieve the dissociation constants: first, using the *EC*_50 _(efficient concentration 50) that is the amount of ligand needed to generate occupation of half the receptors at equilibrium. In this case, this amount is *κ *(and therefore 1 in the dimensionless version). Otherwise, we can also use the slope at origin  (also equals 1 in the dimensionless version).

### Over stacked receptors

Spatial correlation of receptors should in itself modify Eq. 2, as the joint probability to find both reactants in the same vicinity is no longer independent for close receptors. Thus, we first propose an extreme case that has an analytical derivation. Let us assume we have *r*_0 _receptors which are divided among clusters of size *n *- there are *r*_0_/*n *such clusters. We will suppose that receptors inside these clusters are so close together that the area in which ligand binding may occur is the same for each receptor of a cluster. In other words, each receptor of a cluster interacts with ligand localized in the exact same portion of the extracellular vicinity, and clusters of size *n *can be seen as receptors with *n *sites. With this assumption, the ODE describing the equilibrium saturation rate of receptors is a special case of equations considering clusters of size *n *as virtual macromolecules with *n *docking sites, as seen in [36-38]. This simple trick allows us to compute the number of sites occupied *c*. Indeed, let us name *C_i _*(*i *≤ *n*) a cluster with *i *sites occupied (*C*_0 _= *R*, *R *being a cluster with no receptors occupied). The lower case letters, *c_i_*, will denote the numbers of clusters *C_i_*. We discard the transitions for more than one site at a time, yielding only constants for transition between *C*_*i*-1 _and *C*_*i *_(*i *≥ 1)(3)

At this point we simply partitioned the number of clusters *r*_0_/*n *by their amount of occupied sites *i *. Therefore the total number of sites occupied (and of bound ligands) will be , since there are *i *occupied sites per *C_i_*.

From this we can derive a set of ODE's that describe the evolution of concentrations of these components, where we can assume a homogeneous medium. At equilibrium, we obtain a very general formula(4)

where we can relate simply the different association/dissociation constants. We assume that a receptor with *i *occupied sites is *i *times more likely to release one of its cognate molecules than a receptor with only 1 site occupied. Indeed, we have *k_i _*= *k*_1 _but *k*_-*i *_= *ik*_-1_, so *κ_i _*= *iκ*_1_. Due to the shared affinity zone, we will assume in this model that the potential to bind a free site will be independent of the number of free sites. Therefore the on rate *k_i _*will be equal to *k*_1 _because it defines the transition from *L *+ *C*_*i*-1 _to *C*_*i *_through binding of 1 ligand to 1 site. This event happens with the same probability as the transition *L *+ *R *to *C*_1_. Then getting rid of the 1 subscript (*κ *= *κ*_1_)

and(5)

with(6)

**Figure 1:**
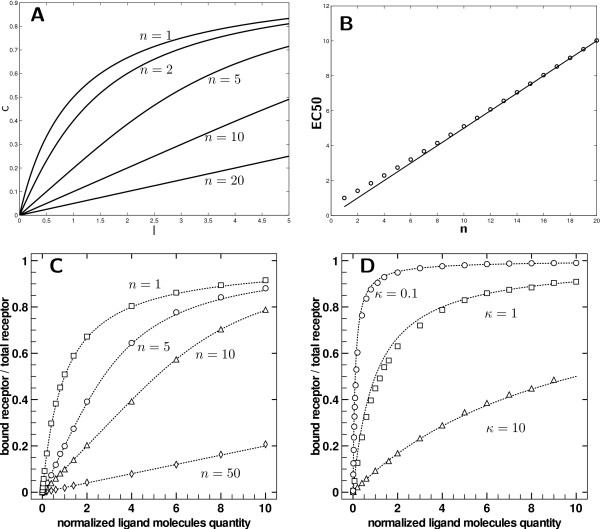
**Model validation and clusters of over stacked receptors**. A) Dose response for reference size *n *= 1 (no cluster) and various cluster sizes *n *∈ {2, 5, 10, 20}. The curves have the same saturation value (*lim c *= 1 when *l *→ ∞). The slope at the origin is 1/*n *and the *EC*_50 _≈ *n*/2. B) Efficient concentration according to the degree of clustering. The line is *EC*_50 _= *n*/2 and the circles are the solution of  using Eq. 4. C) Results for normalized receptor binding with *κ *= 1 and for *n *∈ {1, 5, 10, 50} sites by receptor (respectively squares, circles, triangles, diamonds) compared to theoretical dose response according to Eq. 4 (dashed lines) with same *n*. D) Results of normalized receptor binding for three experiments (circles: *κ *= 0.1, squares: *κ *= 1, triangles: *κ *= 10) compared to theoretical dose responses for respective *κ *according to Eq. 2 (dashed lines).

Several theoretical dose-response (for dimensionless ligand dose  and normalized responses ) curves for different values of *n *are displayed on Figure 1-A.

In the dimensionless case (c* versus l*) the slope at the origin is 1/*n *yielding an apparent affinity of *n*. Even if we cannot simply find the *EC*_50_, we can note that when *n *≫ 1, we can approximate the value by ignoring terms of order greater than one. It first yields that  and  So finally, whenever *n *≫ 1, the dimensionless efficient concentration is(7)

The real *EC*_50 _obtained by numerical computation is compared to Eq. 7 on Figure 1-B. The previous approximation is correct even for low *n*. The very first conclusion to this analysis is that receptor binding dependence can impede or at the least modify dramatically the overall response. Using the same microscopic characteristics (i.e. binding affinity) but with different macroscopic structure, one can create a new *apparent *affinity which is, depending on how it is measured, *n *using the slope or *n*/2 using the *EC*_50_. The local conclusion of this simple analysis is that we can expect modification of the receptor occupation at equilibrium whenever the spatial configuration of the receptors is changed. Introducing correlations in the probabilities of encounter by spatial organization modifies the receptor occupation. In addition, the apparent affinity seems to decrease with the clustering of receptors.

By overstacking affinity zones, even partially, this configuration creates a "strong" spatial correlation which influences dramatically the complex formation rate: within a cluster of receptors, the occupation of a receptor affinity zone is directly dependent of the occupation of affinity zones of the other receptors, since they are totally or partially the same. In order to address the issues stated above, we now propose to investigate what may happen if affinity zones remain distinct from each other inside a cluster of receptors, but "weak" spatial correlation is still induced by placing receptors contiguously. We propose to examine this case using a simulation framework, as no simple mathematical derivation could be obtained.

### Contiguous receptors

We introduce in this section a particle simulation framework that was used to detect the effect of clustering, by modelling clusters of receptors with contiguous but non-overlapping affinity zones. This configuration is taken to be the opposite extreme of over stacked receptors in terms of spatial configuration. That is, within a cluster, receptors are still close to each other, but the presence of ligand in the vicinity of one receptor does not influence the binding of a ligand with receptors of the same cluster: their affinity zones are contiguous.

**Figure 2:**
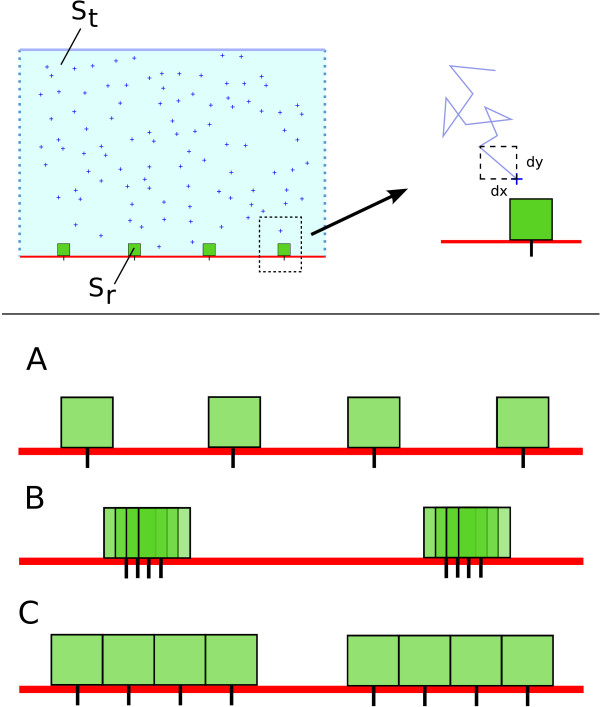
**Simulation environment**. Top panel: On the left is a cartoon view of the 2D membrane of area *S_t_*. Ligand particles are crosses, and the green boxes are receptors (of affinity zone *S_r_*). Receptors are fixed, and ligands undergo a 2D Brownian motion. Bottom panel: cartoon view of the different experiments performed. The spatial configuration of the receptors is modified and the computation of the occupation is performed. Three spatial configuration are tested: A) Evenly spaced receptors - homogeneous repartition. B) Over stacked receptors: the clusters are evenly spaced, but contain a certain number of sites C) Non-overlapping spatial configuration. The affinity zones are contiguous but do not overlap. Clusters of *n *receptors are evenly placed on the membrane.

The simulation is restricted to a 2D environment, and a 1D membrane. Ligands are particles in a 2D environment (see Figure 2). The cell membrane is the bottom segment of this environment. Particles of ligand undergo a 2D Brownian motion in the over-damped regime. Explicitly, using the Euler formalism, the equations of movement are(8)

where *Z_i_*, *i *= 1, 2 are two independent random numbers drawn from a normal distribution of zero mean and variance 1. *D *is the diffusion coefficient and *dt *is the time step for integration. Vertical cylinder boundary conditions are applied for the diffusion; bottom and top segment are bouncing and uncrossable boundaries. The lateral segments are connected: particles that go through one side appear on the other side. To avoid too much transient dependence, initial positions of particles are homogeneous (chosen randomly with uniform probability).

Receptors are punctual but localized only on the bottom line of the environment area. Their diffusion is neglected and they will therefore remain at their initial position throughout the simulations. To simulate docking, we chose a very simple formalism: each receptor has an affinity zone - a square above its position - where there is a constant probability *p*_1 _for a ligand to bind whenever it is found itself in. Of course, a ligand can only bind to a free receptor. No binding event can occur for an already bound receptor. In addition, the bound ligand cannot diffuse as long as it stays bound. Finally, when formed, the complex has a constant probability to dissociate *p*_-1_. Upon dissociation, the ligand molecule resumes its Brownian approximated motion, starting from the center of upper edge of the affinity zone it just left. This is to avoid bias in rebinding events; the probability at the next time step for the ligand to return into the affinity zone or to move away will be equal.

Using this formalism, it is very simple to relate the parameters of the simulation with the association constant of the ligand/receptor binding. Indeed, at equilibrium, the number of receptor-ligand complexes that are dissociating per time step is equal to *p*_-1_*c*.

Assuming the classical framework [5,39], the rate of binding will be the product of three terms: the number of free available receptors - *r*; the probability to find a ligand in the affinity zone - that is *lS_r_*/*S_t _*with *l *as the number of free ligands, *S_r _*and *S_t _*the surface of the affinity zone and the environment respectively; and finally the probability to bind - *p*_1_.

This produces the relation (since what comes out must be equal to what comes in at equilibrium), and using *r *= *r*_0 _- *c*

to obtain the classical equation:

with(9)

Eq. 9 allows a direct comparison with the dissociation constant. It relates simply with docking and undocking probability plus what we called before the affinity zone: the surface available for binding.

## Results

Unless otherwise specified, the parameters are identical for all simulations. The simulations were performed for a sufficient number of time steps to ensure equilibrium was reached, which is around 10^3 ^for the selected parameters. The number of receptor is fixed and is *r*_0 _= 500. Similar runs were performed with *r*_0 _∈ {1000, 2000, 5000, 10000}, showing no qualitative or quantitative differences with *r*_0 _= 500. Thus, the latter value for *r*_0 _was chosen to limit finite-sized effects and computational time. The time step *dt *is equal to 10^-2 ^and *D *= 1. All the results displayed below are normalized on the × axis (ligand molecules) with respect to a reference dissociation constant *κ *= 5.10^5 ^(using a space ratio *S_T _*= 5.10^5^*S_R_*) by taking a constant ratio *p*_-1_/*p*_1 _= 1 with *p*_1 _= *p*_-1 _= 0.1. The results obtained would have to be considered within the correct regime of reaction, that is reaction-limited or diffusion-limited. As the simulated reaction is either one or the other possibility, results cannot be interpreted in the same way. Our concern being the effect of the spatial organization of receptors on binding at equilibrium, we would like to make sure that we simulated ligand-receptor binding in the diffusion-limited regime, so the observation of an effect of clustering can specifically be related to diffusion and geometrical aspects. In order to check whether the simulations were reaction-limited or diffusion-limited, we compared the average mean first passage time (MFPT) of a ligand molecule in a receptor affinity zone to the reaction time-scale.

A diffusion time scale several orders of magnitude larger than the reaction one characterizes diffusion-limited reactions. An estimation of the average MFPT can be obtained using the asymptotic formula from [40] for *r*_0 _traps of surface area *S_r _*which are located on the boundary of a 2D medium of surface area , and gives for our standard set of parameters a MFPT value of approximatively 418. Using the same simulation environment, we also computed first passage times (FPT) of ligand molecules to receptors. The experimental mean first passage time was obtained by non-linear regression of an exponential probability density function with these simulated first passage times. It yields an MFPT estimate of 1267 ± 18 time steps. Both these estimations being consistent and far larger than the reaction time scale, the following results are valid in the context of diffusion-limited reactions but their significance cannot be assured in the reaction-limited case, which would require a dedicated and separate study.

Finally, the number of occupied sites at equilibrium is computed throughout all simulations, and displayed normalized with respect to *r*_0 _= 500.

### No spatial correlation: homogeneous receptor distribution

In the case of evenly distributed receptors (see Figure 2-B top for a cartoon of possible configurations, and Figure 1-D for measurements of receptor occupation), the simulation framework behaves as expected. In particular, the behavior of the particles system is consistent with Eq. 2 and *κ *following Eq. 9 (in the Models section presented above). Three different values for *κ *are used; *κ *= 1 is the reference simulation (*κ *= 5.10^5^, *p*_1 _= *p*_-1 _= 0.01). The two others values for *κ *are *κ *= 10 (using *p*_-1 _= 0.1 = 10*p*_1_) and *κ *= 0.1 (using *p*_-1 _= 0.001 = *p*_1_/10). The results for the several runs are displayed on Figure 1-D. The dashed lines are curves according to the theoretical function (Eq. 2 using the numerical values of the simulation parameters *S_r_*, *S_t _*and the binding properties).

To obtain a good approximation of the slope at origin and the *EC*_50_, more runs were necessary for low concentrations and for values near expected the *EC*_50 _(i.e 1, 0.1). But, all in all, the minimal number of runs is 10 for any given concentration and parameters set. Due to their smallness, error bars are actually negligible - the radius of data points is larger.

As the figures show it and for each parameter set tested, the particles simulation framework is consistent with the predicted behavior: a curvilinear Michaelian-type curve with the correct affinity *κ *- using the simulation parameters *S_r_*, *S_t_*, *p*_1 _and *p*_-1_).

### Over stacked receptors

Spatial correlation in the case of receptors with stacked affinity zones - Figure 1-C - is also checked with the analytical formula Eq.5. Here again, using the predicted affinity *κ *is consistent with the theoretical formulation, as the Eq. 5 is mathematically equivalent to Eq. 2 for *n *= 1.

Three degrees of spatial correlation implied by over stacked receptors (*n ∈ *{1, 5, 10, 50}) are investigated and compared to the control case *n *= 1. Note that the control is of course the same for *κ *= 1 on Figure 1-D. Results are averaged values for five runs (Figure 1-B circles). The dashed lines are theoretical values obtained via Eq. 5. Here again, simulations perfectly match the theory in all cases.

Simulations were in perfect agreement with the mathematical derivations presented in the Models section for both type of layouts (as in Figure 1). Simulations of evenly dispatched receptors follows the classical Ligand-Receptor binding equilibrium equation. When over stacked in clusters of various sizes, the proposed equation 5 and the simulations match. Simulations for the latter case will act as a worst case scenario for clustering of receptors. Indeed, this will be the worst situation as regards to affinity zone availability. It should be expected therefore that the ligand receptor binding would be overlap-dependent. The overall binding should increase as the affinity zone is made available and the overlap is decreasing. The maximal effect would therefore be operating for contiguous but non-overlapping affinity zones.

### Contiguous receptors

**Figure 3:**
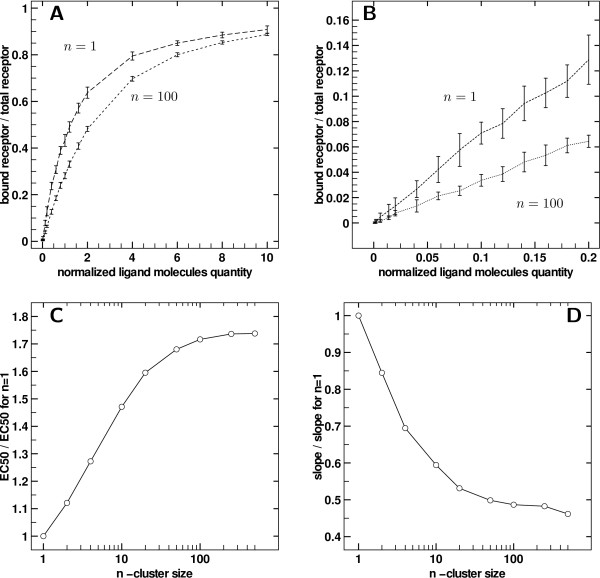
**Effect of clustering for contiguous receptors**. A) Dose response for *n *= 1 (control) and *n *= 100 receptors per cluster. Error bars are ± standard deviation. B) Close-up of A for *l *≤ 0.2. Error bars are ± standard deviation. C) Ratio of fitted *EC*_50 _to control *EC*_50 _(i.e. for *n *= 1) with increasing cluster size, with contiguous receptors, in semi-logarithmic scale. D) Ratio of fitted slope at origin to slope at origin for *n *= 1, with increasing cluster size, with contiguous receptors, in semi-logarithmic scale.

We present in Figure 3 the results of the dose response curves using the third layout - adjacent receptors whose affinity surfaces do not overlap within a cluster.

The dose response curves are compared, all other parameters being equal, to the control case where receptors are homogeneously spread. In Figure 3-A, a comparison of two experimentally obtained dose response curves is displayed. The number *n *refers to the number of receptors per cluster, the total number of receptors remaining equal to *r*_0 _= 500. So *n *= 1 refers to no clustering and is the Michaelian dose response Eq. 2, and *n *= 100 refers to clusters of size 100 (as defined in Figure 2-B). Figure 3-A and 3B thus show how response is modified by clustering: the *EC*_50 _has increased and the response always lies below the control one, in a weaker but similar way than in the over stacked case seen previously.

Figure 3-B is a close-up view of the origin of the Figure 3-A graph. The slopes at origin clearly differ. The apparent dissociation constant computed from the start of the curve is greater in the clustering case, showing strong clustering effect at low ligand concentrations. For all clusters sizes, the slope at the origin as well as the *EC*_50 _can be estimated respectively by linear regression and non-linear least square fitting. For the slopes at origin, simple linear regressions of occupation rate against dose were performed, using values between 0 and 0.05*κ*. On the other hand, *EC*_50 _were estimated by fitting data using Hill functions - a widely used model for non-Michaelian kinetics . The parameters to be adjusted are *κ *and *α *yielding an estimate of *EC*_50_.

*EC*_50 _and slope at origin obtain via fitting are displayed in Figure 3-C and Figure 3-D respectively as a function of cluster size *n *in semi-logarithmic scale. For both parameters and for all cluster sizes, the values are normalized by the control case (*n *= 1).

The graph Figure 3-C shows that *EC*_50 _gradually increases with cluster size until a plateau is reached at around 170% of the control value. Similarly the slope at origine decreases down to 50% of the control value. Observing dose response curves from similar experiments, but with increasing cluster size, leads to observing different affinities for the ligand for receptors at a global scale, whereas the intrinsic affinity of each individual receptor remained equal. The saturation at high cluster sizes is merely due to the fact that no more clustering can be induced once extreme cluster sizes are reached, which are limited by the fixed number of receptors.

The Hill coefficient *α *is classically considered as a reflection of cooperativity in enzymatic reactions. In our case, we observed an increasing *α *with cluster size until saturation under 20% (data not shown). One can note that Hill function is not an appropriate qualitative model for the curves obtained, as slopes at origin are non-zero, but in our case it merely serves as a mathematical support for *EC*_50 _estimation. The very slight variation of Hill coefficient can hardly support any qualitative or quantitative conclusions about clustering effect in the contiguous receptors case, as the Hill function is not pertinent here as a mechanistic model.

### Clustering enhances response by increased rebinding

Intuitively, receptor clustering should induce two opposite effects that counter themselves: enhanced rebinding to close receptors, but decreased ligand-receptor encounter probability. In other words, when receptors are clustered, ligands spend on average more time diffusing before encountering a receptor. Indeed the membrane is not evenly covered and has large receptor-free zones. On the other hand, once bound a ligand will be released in a richer receptor area when receptors are clustered thereby allowing a greater rebinding probability. In order to explore the effect of this rebinding, we perform the following experiment: instead of releasing a ligand at the edge of its former cognate receptor affinity zone when it undocks, the ligand is relocated randomly within the entire medium.

By imposing this random repositioning of ligands after unbinding, the simulation bypasses the potential effect of rebinding, as ligands are on average reinjected quite far from the membrane.

Receptor occupation is then only caused by spatial and temporal independent complex formation. Comparison between dose response curves in such a case and standard simulations may then qualitatively illustrate the part of response alteration which is only due to clustering-enhanced rebinding.

**Figure 4:**
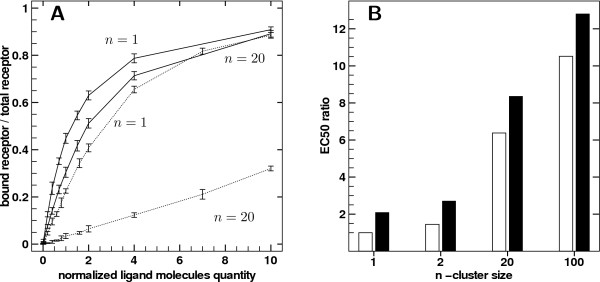
**Effect of rebinding on receptor occupation at equilibrium**. A) Comparison of dose response curves between *n *= 1 and *n *= 20 when ligand is dropped at the edge of affinity surface when unbound (solid lines - standard simulations) or ligand randomly reinjected in bulk when unbound (dashed lines). B) Black bars: ratio obtained with the same layout but using *E*_50 _obtained with random reinjection normalized by *E*_50 _obtained with normal reinjection. White bars: ratio obtained for random reinjection using *EC*_50 _computed for various cluster sizes normalized by *E*_50 _with no clusters (*n *= 1).

Dose response from such simulations are compared with the standard simulations presented so far i.e. the simulations described in the previous section) for the same clustering (i.e. same *n*), in Figure 4.

As mentioned above, the effect of random reinjection strongly affects the receptor occupation even in the unclustered case. Since black bars are increasing with clustering, removing rebinding events has a stronger importance the more the receptors are clustered. It was expected since ligands have a higher probability to rebind when receptors are available in the vicinity. Moreover white bars show that the impact of clustering can be greatly increased via random reinjection when normalized by unclustered case (up to ten times the *EC*_50 _as compared to results in Figure 3-C). In that case the forward rate decrease observed via clustering is not counterbalanced anymore by the greater rebinding dynamics of the clusters. This experiment showed that the decrease in the forward rate due to clustering is stronger than the rebinding gain obtained with closer nearby receptors.

### Clustering through partially overlapping receptors

**Figure 5:**
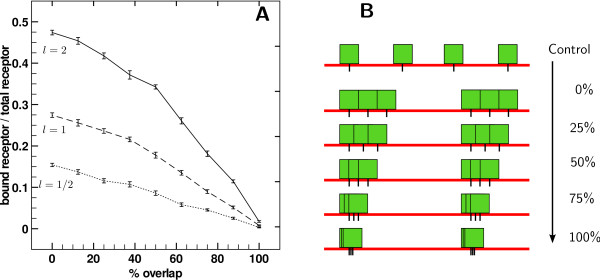
**Receptor occupation when affinity surfaces partially overlapped within a cluster**. A) Comparison of occupation as a function of relative overlap of affinity surfaces. On a single curve, points correspond to the same experiment, for a fixed ligand concentration, but with varying overlap. Error bars are ± standard deviation. B) Cartoon representing increasingly overlapped receptor affinity surfaces within clusters.

Between clusters of over stacked receptors and clusters of adjacent receptors, we investigate an intermediate scenario, in which clusters are composed of receptors with partially overlapped zones. Responses are computed for a single dose *l *∈ {0.5*κ*, 1*κ*, 2*κ*}, with clusters of *n *= 100 receptors progressively overlapping, as the cartoon Figure 5-B pictures. Figure 5-A displays the fraction of occupied receptors at equilibrium in function of intra-cluster overlap, each line corresponding to a given dose *l *as mentioned above.

As the overlap increases, at fixed number of receptors set in a fixed number of clusters, the effective surface covered by receptors decreases, and so decreases the receptor occupation at equilibrium, from 0% to 100% overlap within a continuum. When in clusters, receptors can possibly share a common affinity zone with some of its neighbors. The decreases in apparent affinity is therefore more pronounced in that case. A similar behavior was observed for each cluster size tested.

### Spreading of receptors

**Figure 6:**
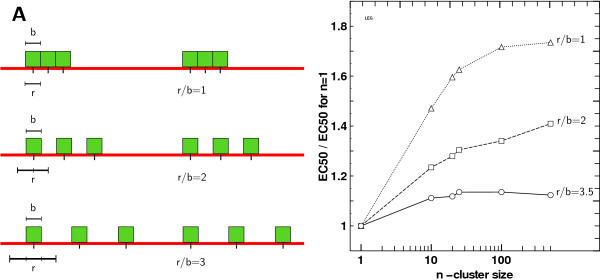
**Receptor spacing, affinity zone size and clustering**. A) Cartoon representing clusters of receptors with different receptor size width (r) on affinity zone width (b) ratio. A fixed affinity zone as it was used in simulation with a increasing receptor width leads to an increasing r/b ratio and therefore to sparser clusters of receptors. B) Ratio of fitted *EC*_50 _to control *EC*_50 _(i.e. for *n *= 1) with increasing cluster size, with contiguous receptors and with respect to receptor width on affinity zone width ratio. The scale is semi-logarithmic.

On the other side, we simulated situations where the affinity zone width (*b*) remained constant but the distance between receptors *r *increased. This could represent a situation where the receptors are still clustered but use a larger space than their binding radius. This layout is depicted on Figure 6-A. We tested two values for the ratio *r*/*b *with *r *>*b*. Note that previously *r*/*b *was always ≤ 1 with equality occurring in the contiguous case. Figure 6-B displays the impact on *EC*_50 _ratios compared to control (for *n *= 100). The effect of clustering decreases whenever receptors are farther away inside a cluster. Intuitively, this could have been expected since the total zone covered by the receptors is much wider and counteracts the clustering effect as receptor positions tend to become homogeneous.

### Ligand diffusion

**Figure 7:**
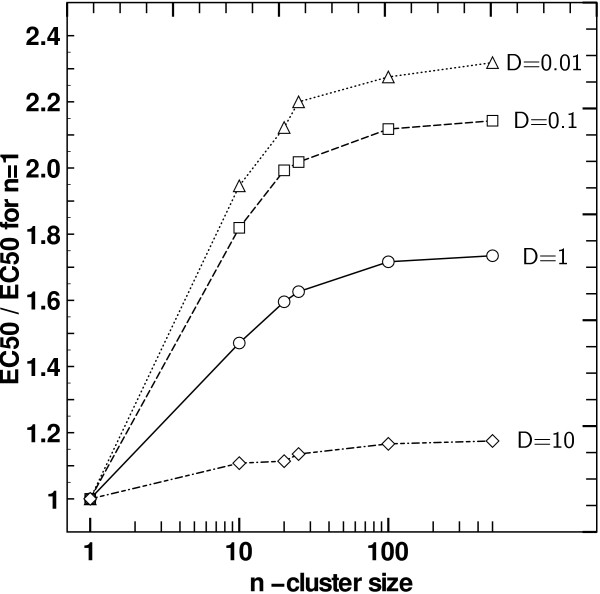
**Clustering effect with different ligand diffusion coefficients**. Ratio of fitted *EC*_50 _to control *EC*_50 _(i.e. for *n *= 1) with increasing cluster size, with contiguous receptors and with respect to the ligand coefficient diffusion used in simulation. The scale is semi-logarithmic.

The simulations were so far performed with ligand diffusion coefficient *D *= 1. Results suggest that the mean time between receptor-ligand encounters is affected by clustering, as receptors positions are correlated, but diffusion itself also affects characteristic times. Simulations were run with diffusion coefficients between 0.01 and 10 (for all the following experiments we used *dt *= 10^-4^), still comparing homogeneous receptor spacing and receptor clustering. After having checked that the equilibrium is reached, we could observe that the receptor occupation in function of the dose decreased, but still reached the same saturation value. We then compared apparent affinities in function of cluster size. Figure 7 shows the comparison of EC50 (obtained via fit) between the clustered and unclustered case. A decrease of *D *yields an amplification of the effect of clustering on response. On the other hand, increasing *D *leads to a much smaller impact on apparent affinities. Slow diffusing ligand molecules will take a longer time to go from a receptor to another than fast diffusing ligand molecules, meaning that two receptors will be "seen" farther from each other by slow diffusing ligand molecules. As expected changing *D *modifies the degree of spatial correlation between receptors, and therefore influences the effect of clustering, as it is only based on the geometry of the system. Spanning three degrees of magnitude of the diffusion does not change the results qualitatively.

## Conclusions

The presented computational model transcribes the necessity of proximity for reactants to interact and combines it with the probabilistic nature of biochemical reactions at microscopic scale. The use of approximated Brownian motion in real coordinates and binding through affinity surfaces in a continuous medium allows the investigation of ligand-receptor reactions at microscopic scale and potentially reduces latent finite size effects of discrete lattices simulations. Modelling receptor as affinity zones with probabilistic binding allows to directly relate simulation parameters with ODE formalism.

Several configurations are explored by means of simulations. First, the model was validated for homogeneous receptor repartition by checking simulation concordance with the classic Michaelian equation. Two extreme cases of clustering were then tested, inducing spatial correlation either considering two possibilities. Within a cluster, receptors could be so close to each other that they interact with ligand particles contained exactly in the same area. Or alternatively, receptor affinity zones could simply be adjacent without overlapping. For receptors with stacked affinity zones, simulations still match the mathematical description.

For contiguous receptors, as no simple mathematical formulation is available, simulations are the only way to explore the potential effect of clustering. Some additional experiments are also performed to study more specifically some local aspects of ligand-receptor interaction, such as rebinding or the effect of partial receptor overlap.

Results suggest some insights about the receptor colocalization effects on ligand-receptor binding, observed on membrane receptors occupation. The ligand-receptor encounter probability is lower when receptors are clustered, because an inhomogeneous membrane covering leads to depleted zones and highly concentrated zones which both contain the same concentration of ligand. Thus, ligand molecules roaming in such depleted zones do not encounter receptors and actual reacting quantities are decreased compared to what is assumed to interact in homogeneous configuration. But, receptor clustering also increases the rebinding probability, in accordance with previous works [32]. These two opposite effects yield a dynamic chemical equilibrium for receptor occupation which differs from the one predicted by reaction rate equation under homogeneous dilution assumption. Simulations suggests that the enhanced rebinding cannot overcome the decreasing effect of spatial segregation and leads to a decreased apparent affinity of the global set of receptors. Nevertheless, the decreasing effect of spatial segregation may be progressively compensated as ligand concentration reaches high levels, since in a ligand-saturated medium, ligand-receptor encounter probability converges to one. Finally, both effects combine in a non-trivial and dose-dependent manner, and give an altered response, which cannot be characterized by the theoretical dissociation constant, and whose shape cannot be described by a classical Michaelian ODE.

Lipid rafts and other membrane structuring components could then serve as signalling modulators by adapting cell sensitivity through receptor clustering. A single kind of receptor could be declined in various apparent affinities by dynamic clustering, and thus be sufficient to give the cell some flexibility in terms of signal response, whereas producing several different types of receptor with different affinities would consume a lot more resources.

Individual-based simulations provide insights into how spatial configuration of complex systems impact the processes they generate. They produce valuable results at both spatio-temporal microscopic scale - e.g. first-time encounter probability, ligand-receptor residence time, average distance travelled between rebinding events distributions - and macroscopic scale, such as receptor occupation at equilibrium, or pharmacodynamic dose-response. Individual-based models also allow for more complete implementations of the biological reality of the studied phenomena. For example, receptor diffusion could be allowed, or receptors could be set in clusters whose size is drawn from pertinent distribution laws, such as normal, exponential or power laws. Simulations would then provide valuable results on the robustness of observed effects of clustering towards realistic and noisy spatial configurations.

Results suggest that receptor clustering has an impact on signalling by itself, without incorporating any specific receptor-receptor interactions in the model. However, it should be interesting to explore specific biological interactions with the model, such as receptor transphosphorylation, hetero/homodimeric receptors or allosteric competition between binding sites, which could be easily implemented and experimented. Simulations could be used to study more complex signalling systems such as G-Protein-based pathways and would inspire useful intuitions for biological experiments, as they provide insights on the functional impact of spatial configurations on the mechanics of signalling.

## Authors' contributions

BC helped to design the study, performed the simulations, analyzed the simulation data and drafted the manuscript. HS conceived the study, analyzed the data and drafted the manuscript. All author read and approved the final manuscript.
